# Minimal Residual Disease in Multiple Myeloma: Something Old, Something New

**DOI:** 10.3390/cancers13174332

**Published:** 2021-08-27

**Authors:** Carlos Bravo-Pérez, María Sola, Raúl Teruel-Montoya, María Dolores García-Malo, Francisco José Ortuño, Vicente Vicente, Felipe de Arriba, Andrés Jerez

**Affiliations:** 1 Hematology and Medical Oncology Department, University Hospital Morales Meseguer, IMIB, 30003 Murcia, Spain; cbravo@alumni.unav.es (C.B.-P.); mss50j@AD.SMS.CARM.es (M.S.); mdgarcia2@ucam.edu (M.D.G.-M.); fortunog@sehh.es (F.J.O.); vicente.vicente@carm.es (V.V.); farriba@um.es (F.d.A.); andres.jerez@um.es (A.J.); 2 CB15/00055-CIBERER, 30003 Murcia, Spain

**Keywords:** multiple myeloma, minimal residual disease, novel agents

## Abstract

**Simple Summary:**

Currently, response rates in multiple myeloma (MM) have increased dramatically, with more than 50% otablef those who respond satisfying complete response criteria. Achieving frequent deep responses has necessarily led to test conceptual advantages for assessing and treating MM patients with only minimal residual disease (MRD). In this review, we present and discuss the clinical relevance, methodology, and challenges for measuring MRD in MM.

**Abstract:**

The game-changing outcome effect, due to the generalized use of novel agents in MM, has cre-ated a paradigm shift. Achieving frequent deep responses has placed MM among those neoplasms where the rationale for assessing MRD is fulfilled. However, its implementation in MM has raised specific questions: how might we weight standard measures against deep MRD in the emerging CAR-T setting? Which high sensitivity method to choose? Are current response criteria still useful? In this work, we address lessons learned from the use of MRD in other neoplasms, the steps followed for the harmonization of current methods for comprehensively measuring MRD, and the challenges that new therapies and concepts pose in the MM clinical field.

## 1. Introduction

Although multiple myeloma (MM) remains an incurable disease, median overall survival (OS) for newly diagnosed patients has dramatically improved in the last few decades, and it is now closer to 10 years, with increasing proportions of long-term survivors [1]. This substantial progress is mostly due to a number of effective therapies, most of them fulfilling the “targeted agent” definition, an increased knowledge of how to best combine them, and better supportive care. However, this OS gamechanger effect has ushered in another paradigm shift. Traditionally, MM was grouped among neoplasms, such as metastatic carcinomas, in which therapy rarely induced complete remissions (CR) [2,3]. Due to the generalized use of proteasome inhibitors, immunomodulatory drugs, and monoclonal antibodies, response rates have also dramatically increased, with virtually all patients responding to therapy and more than 50% of those who respond satisfying CR criteria [4]. Achieving frequent deep responses has necessarily led us to test the conceptual advantages for assessing and treating MM patients with only minimal residual disease (MRD), as less malignant cells potentially would: (i) reduce the possibility of subclonal resistance, (ii) avoid remodeling microenvironments inducing chemoprotective niches, and (iii) allow drug tolerability by adjusting doses or avoiding dose-limiting end organ damage caused by a higher disease burden [5]. This paper will assess this seemingly unavoidable assembly between MM and MRD by addressing the following questions: where do we come from, where are we, where are we going, how we do it, and whether it is affordable. For that purpose, we will follow, as it is not uncommon in our hematology field [6,7,8], a Victorian wedding rhyme, with the poetic license allowing us to alter the order of the “Something blue” and “Something borrowed” verses.

## 2. Something Old: MRD and MM Past

Building evidence showing us that targeting MRD can prevent relapse in cancer comes from experience using adjuvant therapy for epithelial tumors and sarcomas, which has the central aim of eradicating MRD that escapes surgical resection. Studies across multiple solid tumor types have confirmed that more patients achieve long-term disease-free survival with the combination of surgery and adjuvant therapy than with surgery alone [9].

For blood cancers such as acute leukemias or high-grade lymphomas, a single cycle of intensive chemotherapy can induce CR, but virtually no patient is cured without additional therapy to eradicate MRD [10]. Arguably, the most validated application of MRD-directed therapy is in children with acute lymphoblastic leukemia, where tailoring consolidation therapy based on MRD load is now routine practice [11]. The success of this approach relies on directing the intensity of therapy. The next step was established from a chronic myeloid leukemia (CML) scenario, where patients with inadequate initial responses to tyrosine kinase inhibitors (TKIs), or an increase in the level of MRD following an initial response, are commonly tested for the presence of BCR–ABL kinase domain variants that confer TKI resistance, using MRD for targeted approaches to eradicate resistant clones [12]. In addition, patients with CML who achieve optimal responses to first- or second-line TKI therapy (defined as sustained, deep remissions of greater than 4–5 log10 reductions in BCR–ABL transcript using the International Scale) may be candidates for drug cessation after multiple years of TKI therapy, showing us the capacity of MRD to guide therapy discontinuation [13].

Response assessment in MM has traditionally been based on the evaluation of serum and urine monoclonal protein concentrations by means of protein electrophoresis and/or immunofixation, as a surrogate for disease burden, allowing for the detection of minimal amounts of paraprotein [14]. The initial definition of a CR only required less than 5% of plasma cells in the bone marrow (BM), irrespective of their clonal nature. This definition was further refined to stringent CR by the addition of the serum-free light chain ratio assay plus immunohistochemical clonal assessment on the trephine biopsy [15]. These consensus criteria were uniformly incorporated into clinical trials, allowing improved comparisons, and they remained applicable while older therapies were predominant, including autologous stem-cell transplantation (ASCT) when less than half of patients achieved CR [16].

Indisputably, the achievement of MRD negativity conferred a more favorable outcome for treated MM patients, even when the targeted era was making its entrance. A first meta-analysis by Landgren et al. [17], and the one that followed by Munshi et al. [18], which included a higher proportion of studies with “older” therapies and various approaches for MRD detection, verified the prognostic impact of MRD negativity in clinical outcome. The latter meta-analysis showed a 59% reduced risk of progression and 43% reduced risk of death for MRD-negative patients with a median progression free survival (PFS) of 54 vs. 26 months and a median OS of 98 vs. 82 months for MRD-negative vs. MRD-positive patients, respectively. When compared with other prognostic factors, MRD has been shown to be superior and the most relevant predictor of clinical outcome. In multivariate analyses, the achievement of MRD negativity is proven to be the strongest independent prognostic factor, surpassing other favorable prognostic parameters [19].

However, in the last decade, with new therapies targeting the myeloma cell itself, as well as the microenvironment and the immune system, achieving MRD states is now highly feasible [20]. Thus, in 2016, the International Myeloma Working Group (IMWG) defined the presence of MRD in myeloma as having one tumor cell in at least 10^5^ normal cells in BM (minimum sensitivity threshold of 10^−5^), recognized its detection as an important endpoint, and updated the response criteria to include measurement of MRD as the deepest level of response that could be achieved [14]. Since then, using sensitive methods, superior PFS and OS outcomes were observed in patients who achieved MRD negativity [21,22,23].

## 3. Something New: MRD and MM Present and Future

Recently, that prognostic value of MRD in MM has been confirmed as more patients are treated with new agents, and their outcomes are pooled and updated. In 2020, Munshi et al. performed the largest and most recent systematic review and meta-analysis on this issue [24]. They compiled data of 8098 patients from 44 studies published from 1998 to 2019 on the role of MRD in survival outcomes. Twenty-nine studies assessed MRD status by flow cytometry [19,25,26,27,28,29,30,31,32,33,34,35,36,37,38,39,40,41,42,43,44,45,46,47,48,49,50,51,52,53], eight by qPCR [28,54,55,56,57,58,59,60], and nine by next-generation sequencing (NGS) [61,62,63,64,65,66,67]. Some studies evaluated MRD simultaneously by both immunophenotypic and molecular methods. The sensitivity threshold among works ranged from 10^−4^ to 10^−6^.

Overall, 3111 patients were MRD-negative and 4987 were MRD-positive. Cases that achieved MRD-negative status had significantly increased PFS and OS than those who remained MRD-positive, with pooled estimated relative risk reductions for progression and death of 67% (95%CI: 63–71%) and 55% (95%CI: 49–61%), respectively. Subgroup analysis revealed that the prognostic benefit of MRD negativity was independent from the method of MRD evaluation or the sensitivity threshold used. It was also associated with significantly improved survival outcomes, regardless of cytogenetic risk (high or standard risks), the depth of response at MRD assessment (CR and above or very good partial response [VGPR] and above), and the time of MRD measurement (newly diagnosed or relapsed/refractory disease, and before or after maintenance therapy initiation). In summary, cumulative evidence confirms the robust association of MRD status and survival outcomes in MM. Thus, it is rational that numerous ongoing clinical trials are currently considering MRD negativity as an additional primary endpoint.

More recently, the therapeutic landscape of hematologic neoplasms has been revolutionized by diverse targeted agents, such as TKIs and immunotherapy, which are showing high antitumor efficacy, even in patients with unfavorable biological features or refractory disease. Some of the advancements in MM are certainly related to the introduction of newer generations of proteasome inhibitors and immunomodulatory drugs [29,68], but also to novel monoclonal antibodies, combined in potent triplets/quadruplets in early lines of therapy. Daratumumab is the first-in-class anti-CD38 monoclonal antibody used in MM. After demonstrating a strong efficacy in monotherapy or in combination regimens in patients with relapsed/refractory disease [69], it was quickly added to the backbone of early multidrug schemes in numerous clinical trials [65,70]. All of these studies showed that the incorporation of daratumumab yielded higher and deeper rates of response, including a significantly increased proportion of patients achieving MRD-negative status, which was at least doubled in the experimental arm of most trials [71].

These unprecedent responses observed with triplet/quadruplet regimens have resulted in more prolonged survival, but most patients eventually develop refractory disease to one or more drugs from the three aforementioned therapeutic categories, ultimately becoming triple-/penta-refractory [72,73]. For these heavily pretreated and aggressive cases with very poor prognosis, antigen-directed immunotherapies, such as monoclonal/conjugate antibodies [74], T-cell engagers [75], and, notably, chimeric antigen receptor (CAR) T-cell therapy, show impressive outcomes [76]. Unlike kinase inhibitors used in chronic lymphocytic leukemia and B-cell lymphoma, which were themselves associated with potent effect but persistent detectable disease, requiring continuous treatment [77], immunotherapy for MM has the capacity to reach deep clinical responses and MRD negativity [78].

**Figure 1:**
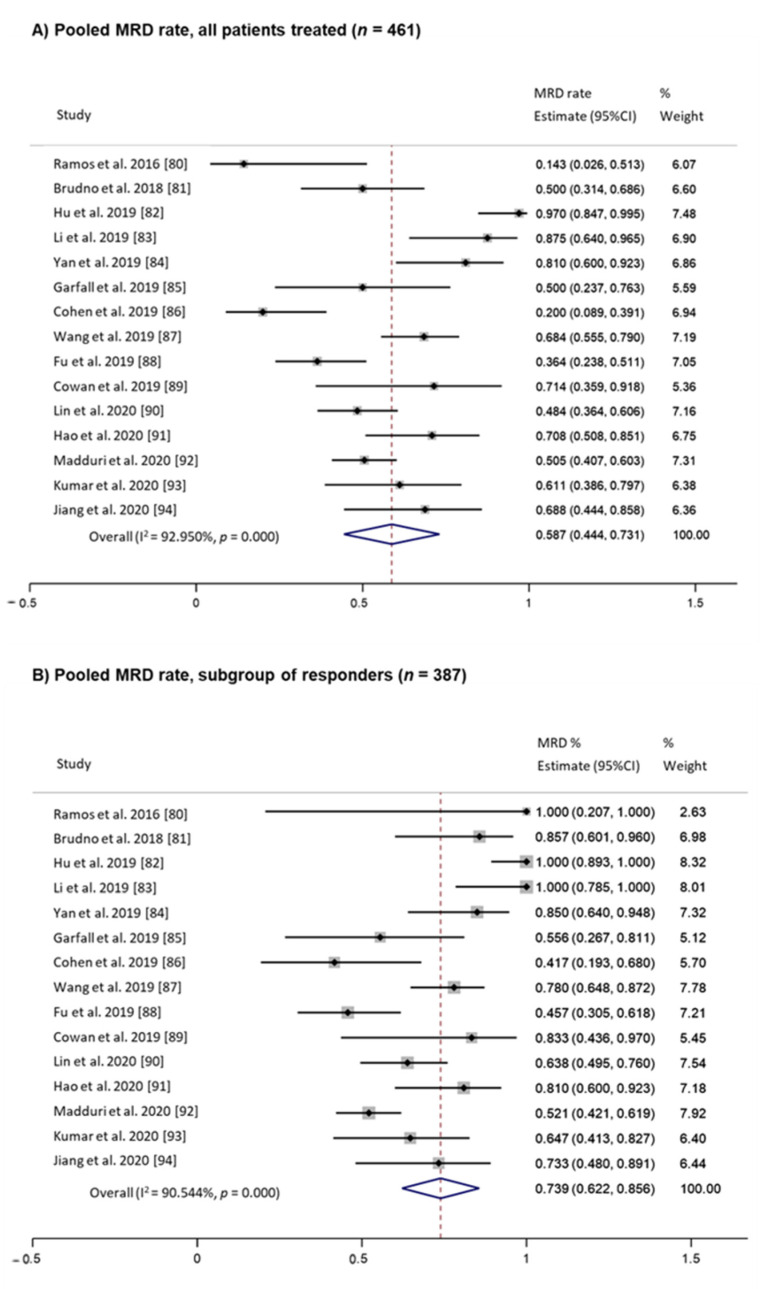
Pooled MRD rate for CAR T cell therapy in MM: (**A**) estimate in all patients treated (*n* = 461). (**B**) Estimate in responder subgroup (*n* = 387). Data were extracted from Mohyuddin et al. meta-analysis [79]. Random effect model by using metaprop command in Stata 16/IC software was performed.

In 2017, Mohyuddin et al. published a systematic review and meta-analysis of 950 MM patients from 30 studies conducted from 2016 to 2020 on CAR T-cells, mainly directed against the B-cell maturation antigen (BCMA) [79]. Since the number of studies with a follow-up long enough for reporting survival outcomes is limited, pooled information regarding PFS and OS is not provided; however, the authors report a summary estimated response rate of 78.3% (95%CI: 72.4–84.3%). In this systematic review, MRD assessment data were collected from 461 patients from 15 studies [80,81,82,83,84,85,86,87,88,89,90,91,92,93,94]. Although pooled MRD analysis is not performed in this work, based on the information provided, we have conducted an estimation in this cohort by using a random effect model, using *metaprop* command in Stata 16/IC software (StataCorp LLC, USA). With this approach, the pooled proportion of patients achieving MRD-negative status estimate was 58.7% (95%CI: 44.4–73.1%) in all patients who were treated with CAR T-cell constructs, and 73.9% (95%CI: 62.2–85.6%) in those patients who had any grade of clinical response (Figure 1). A more prolonged follow-up is needed; however, despite high overall response rates and deep remission, a significant proportion of patients eventually relapse, so strategies to enhance CAR T-cell response durability are necessary, and MRD assessment with increasingly higher sensitivity methods should be guaranteed in future trials.

In addition, new relevant definitions are emerging regarding MM MRD. Using the ongoing POLLUX and CASTOR studies in relapsed/refractory patients, an exploratory analysis has found that “Sustained MRD negativity” (defined as the maintenance of MRD negativity in bone marrow confirmed ≥ 6 or ≥12 months apart) is associated with improved PFS compared with patients who obtain MRD-negative status but not MRD durability [95].

## 4. Something Blue (Reliable): Methods for BM MRD Assessment

Currently, the most common methods for BM MRD assessment are multiparametric flow cytometry (MFC) and high throughput/next generation sequencing (NGS), based on their reliability; these methods can identify disease in more than 90% of cases at a limit of detection of at least 10^−5^ [94].

Thus far, MFC has been the most widely used method for MRD assessment in MM due to its widespread availability, hours-turnaround time, and relatively low cost. In addition, and in contrast to NGS, MFC provides information on BM cellular status and, with recently improved strategies, a disease-baseline analysis is not mandatory. Its main limitations are the lack of standardization among laboratories, a higher dependency on BM sample quality, and the need for fresh samples, preventing a “Send and Hold” approach [96,97].

MFC identifies a myeloma cell by gating those plasma cells (positive for CD138 and CD38) that express aberrantly specific markers (CD56, CD19, CD45, CD27, CD200, CD81, and CD117) and confirms its clonal nature by the restricted expression of intracytoplasmic κ or λ chains [98]. Most laboratories have performed MFC with a limit of detection of 10^−4^, which has been shown to be prognostic even among patients achieving CR [18,99].

Robillard et al. first communicated that their 7-color/8-antibody panel could reach a 10^−5^ sensitivity, one order of magnitude larger than the previous 4-color immunophenotypic investigation of MM MRD (Table 1) [100]. Next, a European consortium, EuroFlow, proposed its two 8-color tube strategy, which is considered the gold standard for flow MRD evaluation due to its high sensitivity (2 × 10^−6^) and high level of standardization regarding processing, acquisition, and data analyzing procedures. It acquires information of 10 specific antigens by means of surface-only and surface/cytoplasmic-staining tubes. An anti-CD38 multiepitope antibody was incorporated in the Euroflow design to prevent the interference of anti-CD38 monoclonal antibodies such as daratumumab [36]. Recently, reassuring results about the feasibility of a complete standardization among different centers were reported from a quality assessment program using fresh material from MM patients within the EMN02/HO95 MM trial involving four reference laboratories willing to commit to EuroFlow protocols [101].

To reduce costs and laboratory labor burden, different approaches testing a single tube strategy have been proposed. Roshal et al. compared the two 8-color tubes proposed by Euroflow with the Memorial Sloan Kettering Cancer Center single surface/cytoplasmic-staining 10-color tube [102]. The latter also gathers information from the 10 same antigens distributed in Euroflow’s tubes [98,103]. The overall concordance between the two tests was 98% and the authors concluded that both fulfilled the FDA-NCI guidelines [104], international consensus recommendations for myeloma flow cytometry-based MRD quality control [105], and the IMWG clinical response criteria for MRD negativity [14]. In fact, both updated versions of MFC have led to further enhancements in prognostication, as recently shown [53,102]. New 8-color single-tube approaches have recently been proposed, both achieving a sensitivity of 10^−5^. First, Duraclone, a streamlined approach for MRD detection in myeloma, showed excellent intra- and inter-assay accuracy. Nevertheless, as the original paper used a ninth antibody (CD117) and the strategy does not include the assessment of clonality, validation efforts recognized it as a good option, with some caution, as some discrepancies may arise [106]. In fact, greater confidence was recently added to the inclusion of light chain antibodies and permeabilization, where data are supported by the identification of a monotypic population with the same light chain as the monoclonal component [107]. Second, which is also as an alternative to more cost-intensive panels, the Freiburg group presented an MM MFC panel, and validated it in cell lines and in routinely assessed MM patients, both in and outside clinical trials [108].

Nevertheless, a common denominator for all groups is the emphasis placed on the quality of the sample as a key factor for MM flow MRD assessment. Not sending the first BM sample pulled or sending them in a different sequence for the same patient through evaluations, will lead to significant hemodilution and variability, in both cases raising the possibility of underestimating actual MRD [109]. As stressed in the recently published consensus effort for MRD performing and reporting harmonization, on a CR MM patient, MRD is the strongest prognostic information that can be acquired from BM aspiration. Thus, in these CR patient evaluations, they advocate for the first BM pull to be sent for MRD testing. If this is not possible, they recommend the next pull to be extracted and sent with a repositioning of the needle to reduce the likelihood of hemodilution [110]. Other strategies, such as functional imaging and liquid biopsy, which may be complementary and useful to resolve false-negative MRD evaluations due to BM sample hemodilution or spatial heterogeneity, are discussed below.

High Throughput Sequencing. The use of NGS in hematology has been a major advance not only in diagnosis and prognosis, but also in the follow-up of MRD in different neoplasms. Speaking of MM, the use of NGS has seen rapid implementation, since it was only in 2011 when the first genetic data on MM emanating from NGS techniques were published [111]; only a few years later, the FDA approved the use of these techniques for the monitoring of MM MRD [112]. Therefore, the implementation of NGS has not only allowed a deeper and more detailed knowledge of MM underpinning genetics, but it has also had a significant impact on the diagnosis and prognosis of the disease by enabling a precise MRD measurement in the BM, either during or after MM treatment.

Currently, for the detection of MM sequencing MRD, the main molecular target is the clonal rearrangement of the immunoglobulin gene. Initially, the evaluation of MRD at the molecular level was assessed by multiplex polymerase chain reaction (PCR) of the *IGH* locus with consensus primers, followed primarily by direct Sanger sequencing [113]. By definition, Sanger sequencing is not the most suitable technique for MRD assessment because of its low sensitivity, as it also amplifies normal B cells, but it must be understood in a historical context, where MRD evaluation was performed at any given time with the most sensitive techniques available. To improve the limit of detection, the allele-specific-oligonucleotide PCR (ASO-PCR) technique, which consists of a real-time PCR with primers and probes specific for a given region of the *IGH* gene, started being used [114]. Compared to Sanger sequencing, ASO-PCR greatly increases the threshold (to 10^−5^), but it is not without limitations. Although it is highly sensitive, ASO-PCR is not a specific technique. Since the amplified region is an area in which there is a high rate of somatic hypermutation, it prevents the correct annealing of the probes used in an ASO-PCR assay, allowing the identification of a molecular marker in only 50–60% of patients.

The recent implementation of NGS after PCR with consensus primers, which results in the sequencing of all PCR products, has overcome the aforementioned technical problems, and has resulted in an approach with both high sensitivity (of 10^−6^) and high specificity [115]. According to IMWG criteria, sequencing MRD negativity is defined as the absence of clonal plasma cells (less than 2 identical sequencing reads) on BM aspirate by NGS, using a validated method with a minimum sensitivity threshold of 10^−5^ or higher [14]. Numerous works have not only confirmed that NGS sequencing is a powerful tool for the detection of MRD, but also that sequencing MRD negativity constitutes a strong prognostic factor [47,61,62,63,64,65,95,116].

In conclusion, despite not being as widespread as MFC in clinical practice, NGS is nevertheless a reliable, sensitive, and reproducible technique for MM MRD evaluation, and it will be increasingly implemented in clinical practice.

## 5. Something Borrowed: Functional Imaging and Liquid Biopsy

^18^F-Fluorodeoxyglucose (FDG)-positron emission tomography (PET) was first introduced in the lymphoma field in the early 1990s. Shortly thereafter, multimodal FDG-PET/computed tomography (CT) was shown to overpass FDG-PET and CT when performed separately by combining functional and morphologic data, and it changed patient care. Currently, FDG-PET/CT constitutes the most valuable imaging technique for staging, treatment response assessment, and monitoring of the majority of lymphoid neoplasms. Imaging techniques have played a key role in the diagnosis of patients suspecting plasma cell disorders. However, the transition from classic radiographic skeletal surveys to more modern imaging methods has been more gradual in MM [117].

The skeleton is affected in up to 80% of MM patients at diagnosis, and roughly 10% of them have focal lesions (FL) and extramedullary disease (EMD). Furthermore, an incidence of EMD of 9% during disease course has been reported [118]. With respect to initial diagnostic screening, there has been a consensus since the 2014 IMWG recommendations on using whole-body low dose CT (WBLDCT) over skeletal survey, due to its higher sensitivity [119,120]. Since then, magnetic resonance imaging (MRI) and, mainly, multimodal FDG-PET/CT, have been incorporated into diagnostic routines [121]. Despite positive results supporting BM MRD assessment in MM, plasma cell infiltration normally exerts a patchy pattern, and conventional skeletal screening may overlook up to 20–30% FL and EMD. MRI and PET/CT have been shown to be significant prognostic factors, and they complement BM response assessment. Accordingly, the IMWG 2016 defined the concept of “imaging plus MRD-negative” as the deepest category of response to be achieved, highlighting the relevance of image tools in the evaluation of the depth of response [14].

MRI is a sensitive method for detecting early focal lesions (FL) and diffuse infiltration patterns of the bone marrow by myeloma cells, with an impact on prognosis regarding the number of FL [122]. MRI should be recommended when WBLDCT is negative and a solitary bone plasmacytoma is suspected [123]. However, in terms of response assessment, MRI has shown high-false positive rates, revealing FL that are not necessarily active. New functional MRI techniques have been developed in order to improve MRI results [124]. Diffusion-weighted magnetic resonance imaging (DW-MRI) examines the water movement at a cellular level within tissues. Limited water movements indicate high cellularity, while a rise in motion of water is related to low cellularity. This technique shows a sensitivity of 90% and a specificity of 93%, discriminating myelomatous from normal marrow [125,126]. Recent publications suggest that DW-MRI could be slightly superior to PET/CT in the assessment of the MRD. Furthermore, it has been recently reported a better PFS in patients with MRD negative and complete imaging response by DW-MRI. Concluding that these two techniques may be complementary for the definition of response [127].

FDG-PET/CT has been successfully incorporated in the diagnosis and follow-up of patients diagnosed with MM. Even though FDG-PET/CT has lower sensitivity compared to MRI for the detection of BM involvement, its specificity is higher, owing to its capacity to differentiate viable from non-viable lesions [128]. Additionally, data have been published regarding the prognostic value of FDG-PET/CT at the time of diagnosis and follow-up [129,130]. The IFM2009 trial showed equivalent efficacy for MRI and FDG-PET/CT in the detection of bone lesions at diagnosis. However, MRI did not show a correlation between normalization of images and improvement in PFS, while FDG-PET/CT proved its value regarding response evaluation [131]. Although follow-up assessment remains unclear, concerning image techniques, the IMWG recommends FDG-PET/CT when evaluating MRD status [123]. Tracers other than FDG, such as sodium ^18^F-Fluoride (NaF), choline-based tracers, and ^68^Ga-Pentixafor ligand, targeting CXCR4, are still under investigation. Early data suggest that choline-based PET/CT detects up to 75% more focal lesions than FDG-PET/CT. However, these results will need to be validated in further studies [132]. Regarding CXCR4, its interest is increased due to its potential theranostic applications, due to its role in disrupting the interaction of MM cells with the BM microenvironment [133]. Finally, immunePET is a novel diagnostic approach that is based on therapeutic antibodies targeting specific myeloma antigens. The most relevant is CD38, which is expressed on myeloma cells, and it is used as an anti-CD38 target. This antibody has been radiolabelled (Zr-89 and Copper-64), and it offers radio-immune PET imaging [134]. Studies in this field are needed in order to prove the applicability of these therapies and determine their contributions to MRD assessment.

Currently, the use of imaging techniques in the follow-up in all patients remains unclear, though the IMWG advocate for the use of FDG-PET/CT as a response assessment when evaluating MRD status. The application and interpretation of the results of these new imaging techniques need further evaluation.

A liquid biopsy is defined as the isolation and analysis of circulating tumor-derived components (mainly cells and/or nucleic acids) from bodily fluids, primarily blood. Developed in the solid tumor field, where accessibility to the tumor cell is quite limited, it has proven its usefulness in different areas such as early detection, risk stratification, and monitoring of response, changes to the tumor clonal dynamics, detection of mechanisms of resistance, and measurement of MRD [135]. Recently, the potentials benefits of a non-invasive technique for the periodic MRD assessment have raised awareness among MM caregivers [136]. The EuroFlow consortium recently reported that MRD, by means of detecting MM circulating cells, was present in 17% of patients in CR, identifying a subgroup of patients with a significant shorter PFS [137]. However, both this MFC and an NGS (detecting Ig gene rearrangements) study found that approximately 40% of patients displayed MRD in BM that was undetectable in PB [138,139], underscoring the need for additional analysis to refine the utility of blood in monitoring MM disease. Notably, assessing the presence of additional genomic markers, such as recurrent mutations and copy number variants, may expand the sensitivity of this approach. Of late, patients with MM have been successfully monitored by targeted sequencing for mutations in key driver genes such as KRAS, NRAS, BRAF, and TP53 [140]. On the other hand, copy number variations are detectable in the vast majority of MM patients, assessed by whole exome sequencing of cfDNA and circulating tumor cells (CTC) [141]. A higher level of cfDNA at diagnosis and a relevant decrease after initiating treatment have been related with poorer survival and prolonged PFS, respectively [142]. As cfDNA contains sequences released from different MM foci, it may be particularly suitable for MM with EMD, where the need for multiple biopsies and the spatial/temporal genomic heterogeneity may prevent precise characterization and monitoring [143].

## *6.* A Silver Sixpence in Her Shoe: MRD Cost-Effectiveness

An abstract presented by Carlson et al. at the 2019 American Society of Clinical Oncology (ASCO) Annual Meeting evaluated the cost-effectiveness of MRD assessment during maintenance treatment for MM patients [144]. Yearly NGS MRD evaluation was compared with no MRD testing. The researchers assumed that maintenance treatment would be stopped when MRD negativity was achieved. They set the cost of MRD assessment at USD 3636 per sample, the cost of maintenance therapy at USD 21,168 per month, and the cost of relapsed disease treatment at USD 27,422 per month. According to their model, MRD evaluation might save a mean of USD 1,156,600 over patient’s lifetime. A second analysis, communicated by the same group at the 2020 American Hematology Association (ASH) Annual Meeting, based on institutional data from an annual cohort of 198 MM patients, estimated an overall annual saving of USD 18,100,000 [145]. They also suggested that MRD assessment led to an improvement in patients’ quality of life due to a lower rate of overtreatment and, consequently, lower risk of treatment-related adverse events than in the group of patients with no MRD testing. Thus, although further works are needed, a risk-adapted maintenance strategy guided by MRD assessment may not only be adequate in terms of clinical efficacy but also favorable in terms of patient utility and institutional monetary benefit.

## 7. Conclusions

Clinical trials have recurrently showed that achieving a deep response with a BM free of residual disease confers a significant and relevant longer survival. The final purpose is to bring MRD to real world clinics (easy to use methods, out of tertiary centers) and use it to guide therapy strategies by “tapering” doses or even through the discontinuation of maintenance treatment for those patients with a sustained negative MRD (it may prove cost-effective).

This will require a large amount of data from the use of MRD in a variety of clinical contexts, which can only be obtained through collaborative efforts. In the targeted agent context, new challenges emerge. For instance, patients who are still in good partial response (VGPR) may achieve MRD negativity in the BM before the monoclonal component is cleared, even at a sensitivity of 10^−6^ [64]. This disengagement of the traditional close parallelism between the monoclonal component and the myeloma cell burden in the BM, or “M-component lag”, is particularly evident in the setting of new immunotherapies, where CAR-T treatment has demonstrated rapid marrow clearance [146]. Thus, recommendations were issued to incorporate MRD testing in patients who achieve VGPR IMWG criteria [147]. Moreover, some voices are already questioning whether the multilayer response assessment is needed in the new agent era. Recent data from the Spanish group demonstrated, in cases with persistent MRD, that no added prognostic value was found by stratifying transplant-eligible MM patients, treated with optimal induction and consolidation, by the current IMWG criteria. Further, they showed plasma cell counts by morphology and serum-free light chain ratios were prognostically useless in patients with a negative immunofixation [148]. Thus, it can be anticipated that the new agent era will create new response criteria, giving more weight to MRD measurements. Current and past efforts on standardization of testing, harmonization of reporting, and incorporation as critical endpoint in clinical trials are solid building blocks for a long-lasting and successful MM and MRD association.

## Figures and Tables

**Table 1:** MM Flow-MRD Panels with a sensitivity, at least, of 10^−5^.

Laser Line	Robillard	Euroflow		MSKCC	Duraclone	Freiburg
Violet	CD38_H450_	CD138_BV421_	CD138_BV421_	CD81_PB_	CD38-nonME _PB_	CyIg λ _AF405_
		CD27_BV510_	CD27_BV510_	CD38_BV510_	CD45_KrO_	CD19_BV510_
				CD27_BV605_		
Blue	CyIg λ _FITC_	CD38ME_FITC_	CD38ME_FITC_	CyIg κ _FITC_	CD81_FITC_	CyIg κ _FITC_
	CD28 + CD56_PE_	CD56_PE_	CD56_PE_	CyIg λ _PE_	CD27_PE_	CD27_PE_
Yellow/ Green	CD138_PC5_	CD45_PerCP-Cy5.5_	CD45_PerCP-Cy5.5_	CD117_PC5.5_	CD19_PC5.5_	CD56 _PerCP-Cy5.5_
	CD19_PE-Cy7_	CD19_PE-Cy7_	CD19_PE-Cy7_	CD19_PC7_	CD200_PC7_	CD38_PE-Cy7_
Red	CyIg κ _APC_	CD117_APC_	CyIg κ _APC_	CD138_APC_	CD138_APC_	CD138_APC_
				CD56_APC-R700_		
	CD45_APC-H7_	CD81_APC-A750_	CyIg κ_APC750_	CD45_APC-H7_	CD56_APC-A750_	CD45_APC-H7_
Sensitivity	10^−5^	10^−6^		10^−6^	10^−5^	10^−5^

MSKCC, Memorial Sloan Kettering Cancer Center; PB, Pacific Blue; KrO, Krome Orange; ME, multiepitope; PE, R-phycoerythin; PC7, PE-cyanine 7; APC, allophycocyanin.

## Data Availability

No new data were created or analyzed in this study. Data sharing is not applicable to this article.
